# APO-9′-Fucoxanthinone Extracted from *Undariopsis peteseniana* Protects Oxidative Stress-Mediated Apoptosis in Cigarette Smoke-Exposed Human Airway Epithelial Cells

**DOI:** 10.3390/md14070140

**Published:** 2016-07-21

**Authors:** Jun-Ho Jang, Ji-Hyeok Lee, Hitendra S. Chand, Jong-Soo Lee, Yong Lin, Nathaniel Weathington, Rama Mallampalli, You-Jin Jeon, Toru Nyunoya

**Affiliations:** 1 Department of Medicine, University of Pittsburgh, Pittsburgh, PA 15213, USA; jangj2@upmc.edu (J.-H.J.); weathingtonnm@upmc.edu (N.W.); mallampallir@upmc.edu (R.M.); 2 Medical Specialty Service Line, VA Pittsburgh Healthcare System, Pittsburgh, PA 15240, USA; 3 Department of Marine Science, Jeju National University, Jeju 690-756, Korea; lee198186@daum.net (J.-H.L.); youjin2014@gmail.com (Y.-J.J.); 4 Lovelace Respiratory Research Institute, Albuquerque, NM 87108, USA; hchand@lrri.org (H.S.C.); ylin@lrri.org (Y.L.); 5 Department of Seafood Science and Technology, Institute of Marine Industry, Gyeongsang National University, Tongyeong 650-160, Korea; legs@gnu.ac.kr

**Keywords:** brown algae, apo-9′-fucoxanthinone, cigarette smoke, airway epithelial cells, DNA damage, apoptosis, oxidative stress

## Abstract

Long-term cigarette smoking increases the risk for chronic obstructive pulmonary disease (COPD), characterized by irreversible expiratory airflow limitation. The pathogenesis of COPD involves oxidative stress and chronic inflammation. Various natural marine compounds possess both anti-oxidant and anti-inflammatory properties, but few have been tested for their efficacy in COPD models. In this study, we conducted an in vitro screening test to identify natural compounds isolated from various brown algae species that might provide protection against cigarette smoke extract (CSE)-induced cytotoxicity. Among nine selected natural compounds, apo-9′-fucoxanthinone (Apo9F) exhibited the highest protection against CSE-induced cytotoxicity in immortalized human bronchial epithelial cells (HBEC2). Furthermore, the protective effects of Apo9F were observed to be associated with a significant reduction in apoptotic cell death, DNA damage, and the levels of mitochondrial reactive oxygen species (ROS) released from CSE-exposed HBEC2 cells. These results suggest that Apo9F protects against CSE-induced DNA damage and apoptosis by regulating mitochondrial ROS production.

## 1. Introduction

Many of the diverse marine organisms are rich natural sources for structurally unique and biologically active chemicals [1]. Recent in vitro studies have identified various biological functions for compounds derived from brown algae, including antioxidant [2], anti-inflammatory [3,4], antibacterial [5], anti-HIV [6], and anti-allergic [7] properties. Such findings suggest that brown algae species may be a novel source of pharmacophores that have the potential to serve as therapy for an array of human diseases.

Cigarette smoking increases the risk for many age-associated diseases, including chronic obstructive pulmonary disease (COPD) characterized by a permanent expiratory airflow obstruction [8]. Airway epithelial cells are among the primary targets for cigarette smoke (CS) exposure and propagate inflammatory responses in COPD. CS contains abundant reactive oxygen/nitrogen species (RONS) and carcinogens, such as polycyclic aromatic hydrocarbons and *N*-nitrosamines [9,10], which induce DNA damage and activate the DNA damage response (DDR) mediated by phosphoinositide 3-kinase related protein kinases (PIKKs). One such PIKK, ataxia teleangiectasia mutated (ATM) protein [11,12], is activated through autophosphorylation at the serine 1981 residue in response to a DNA double strand break (DSB). ATM, in turn, phosphorylates serine 139 of H2AX variant (γH2AX) on chromatin flanking DSB sites, allowing γH2AX, to relay subsequent DDR signaling and DNA repair. As such, γH2AX is widely used as a biomarker for DSBs [13,14]. However, when DNA damage is extensive, the repair pathway is overwhelmed, and the cells may activate mediators of apoptotic cell death [15].

CS or Cigarette smoke extract (CSE) can induce DNA damage and cytotoxicity that can be mediated by oxidative stress. Several in vitro studies demonstrate that some anti-oxidants, such as *N*-acetyl cysteine attenuate CS-induced DNA damage and cell death [16,17]. Mitochondria are an important site of DNA damage [18] and endogenous ROS production in response to CS. Release of toxic ROS and the mitochondrial damage associated molecular pattern molecules (including mito-DNA) critically regulate cell fates including apoptotic cell death [19].

In this study, we evaluate nine natural marine compounds in the context of CSE-induced cellular injury in immortalized human bronchial epithelial cells (HBEC2 cells). Among these, the brown algae derived compound, apo-9′-fucoxanthinone (Apo9F), confers robust protection against CSE-induced DNA damage and cytotoxicity. The protective effects of Apo9F are accompanied by the mitigation of apoptosis, DNA damage, and mitochondria-derived ROS production.

## 2. Results

### 2.1. Apo9F Protects against Cigarette Smoke-Induced Cytotoxicity in Immortalized Human Bronchial Epithelial Cells

**Figure 1:**
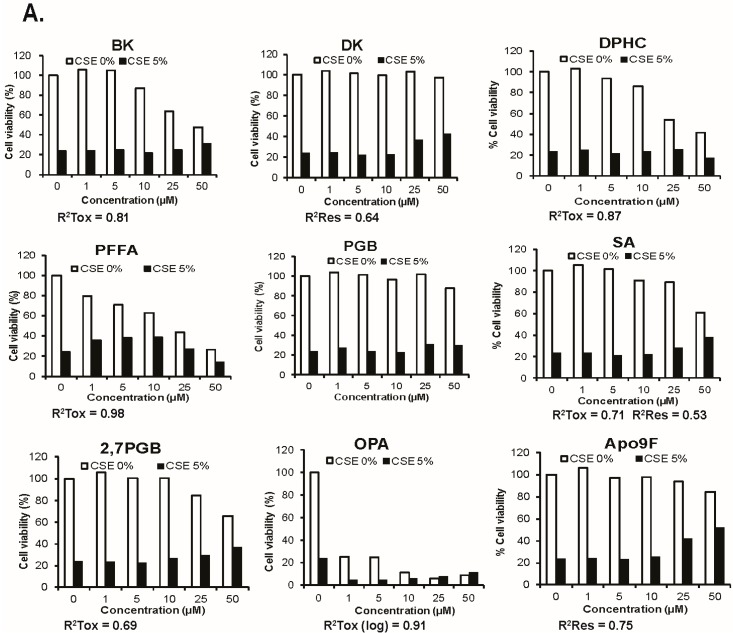

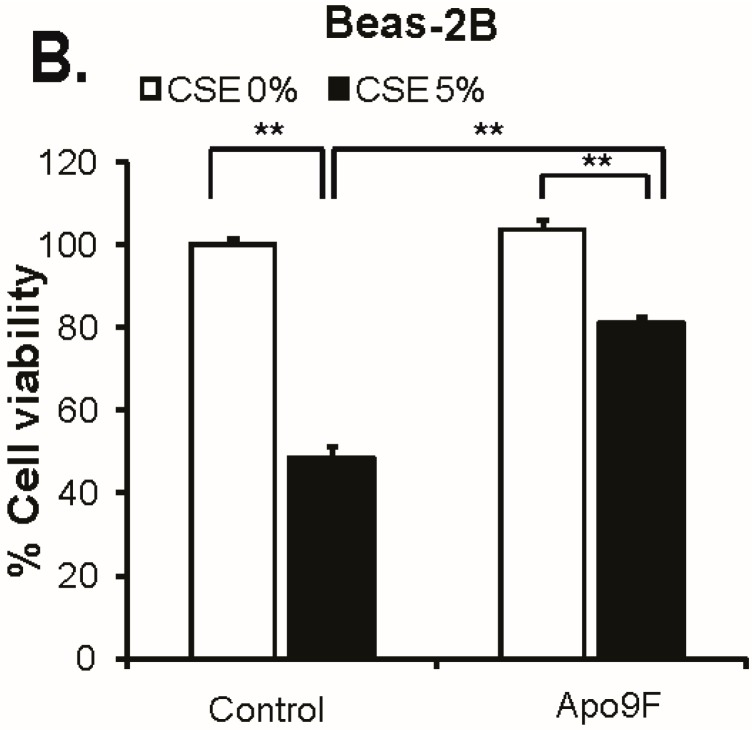
Screening of nine marine compounds for protection against cigarette smoke-induced cytotoxicity in cultured immortalized human bronchial epithelial cells. (**A**) HBEC2 MTT (3-(4,5-dimethythiazol-2-yl)-2,5-diphenyl tetrazolium bromide)-based cell viability after treatment with various concentrations of the nine individual marine compounds (0, 5, 10, 25, and 50 µM in DMSO (dimethyl sulfoxide)) in the presence or absence of 5% CSE (cigarette smoke extract) for 24 h. Single measurements for each data point with *R* values greater than 0.5 are shown for toxicity (0% CSE) or rescue (5% CSE); (**B**) BEAS-2B cells were cultured with 50 μM Apo9F in the presence or absence of 5% CSE for 24 h and assayed for viability. Data are expressed as mean ± SEM (** *p* < 0.01) cytotoxicity in cultured immortalized human bronchial epithelial cells. (**A**) HBEC2 MTT-based cell viability after treatment with various concentrations of the nine individual marine compounds (0, 5, 10, 25, and 50 µM in DMSO) in the presence or absence of 5% CSE for 24 h. Single measurements for each data point with *R* values greater than 0.5 are shown for toxicity (0% CSE) or rescue (5% CSE); (**B**) BEAS-2B cells were cultured with 50 μM Apo9F in the presence or absence of 5% CSE for 24 h and assayed for viability. Data are expressed as mean ± SEM (** *p* < 0.01).

To identify natural marine compounds that protect against CSE-induced cytotoxicity, we conducted an in vitro screening test using the nine compounds isolated from various brown algae species (Table 1). To determine this, we cultured HBEC2 cells with the nine different isolated compounds in the presence or absence of 5% CSE for 24 h, and determined cell viability using the MTT (3-(4,5-dimethylthiazol-2-yl)-2,5-diphenyltetrazolium bromide) assay. In this single screening experiment, some of the tested compounds had a CSE-protective effect on cell viability, while others including phlorofucofuroeckol A (PFFA), octaphlorethol A (OPA), and diphlorethohydroxycarmalol (DPHC) were cytotoxic. These results are presented in Figure 1A with *R*^2^ values that exceed 0.5 presented for trends of the compounds’ cytotoxicity or their rescue from CSE-induced cell death. Among the nine compounds examined, only the dieckol (DK) compound and Apo9F showed protection from CSE- mediated cell death without toxicity, and Apo9F provided the greatest magnitude of protection against CSE-induced cytotoxicity with little intrinsic toxicity. At 50 μM, a 30% increase in viable cells represents a more than doubling of the number of cells surviving CSE exposure compared to the vehicle control. The protective effect of Apo9F on CSE-induced cytotoxicity was next confirmed in another immortalized airway epithelial cell line, BEAS-2B cells, where cells treated with Apo9F again exhibited ~60% less cytotoxicity from the CSE challenge (Figure 1B). These results suggest that Apo9F protects against CSE-induced cytotoxicity in cultured human airway epithelial cells, and, therefore, we further analyzed Apo9F with the following experiments.

### 2.2. Apo9F Suppresses Cigarette Smoke-Induced Apoptotic Cell Death in HBEC2 Cells

**Figure 2:**
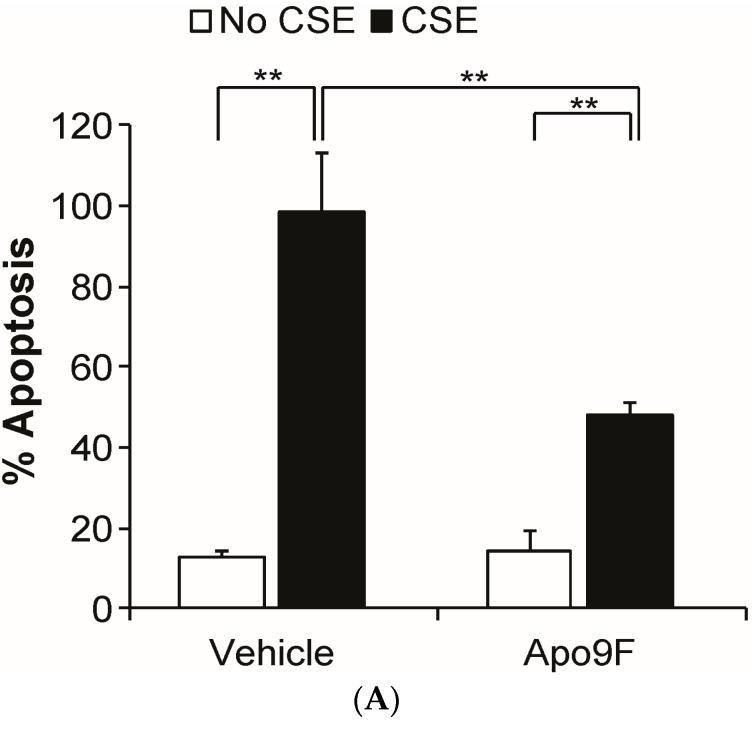

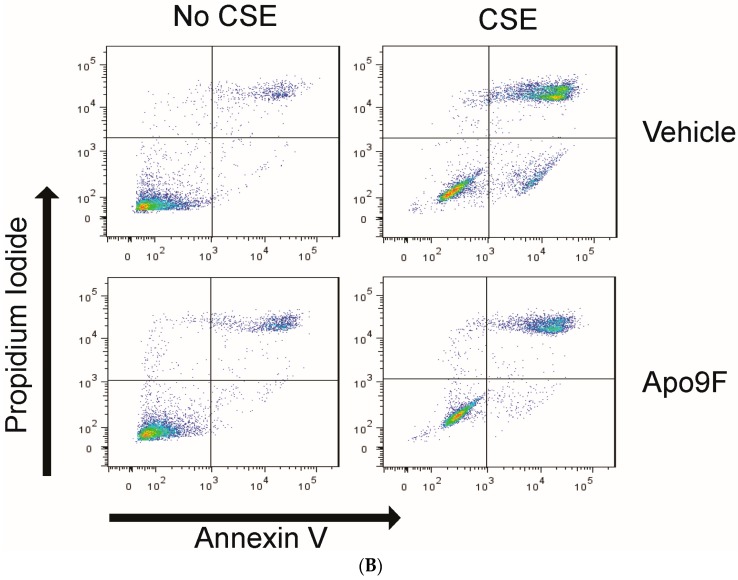
Apo9F suppresses apoptotic cell death in cultured immortalized human bronchial epithelial cells. (**A**) HBEC2 cells were cultured with 50 µM Apo9F in the presence or absence of 5% CSE for 24 h. Cell death was analyzed by Annexin Vand propidium iodide (PI) staining 24 h after CSE exposure. The percentage of Annexin V positive cells/total cell number was expressed as percentage apoptosis. Data are expressed as mean ± SEM for three independentexperiments (** *p* < 0.01); (**B**) representative flow cytometry data are shown.

CS exposure is known to induce apoptotic cell death in cultured HBEC2 cells [26]. We next determined whether Apo9F attenuates CSE-induced apoptotic cell death using a flow cytometric assay with dual staining of Annexin V and PI (propidium iodide). Consistent with the results of MTT cytotoxicity assay, Apo9F attenuated apoptotic cell death with only 40% cells undergoing CSE-induced apoptosis compared to 95% apoptotic cells in the vehicle-treated controls (Figure 2). These data suggest that Apo9F protects against CSE-induced apoptosis in cultured HBEC2 cells.

### 2.3. Apo9F Decreases Mitochondria-Derived ROS Production in Cigarette Smoke-Exposed HBEC2 Cells

**Figure 3:**
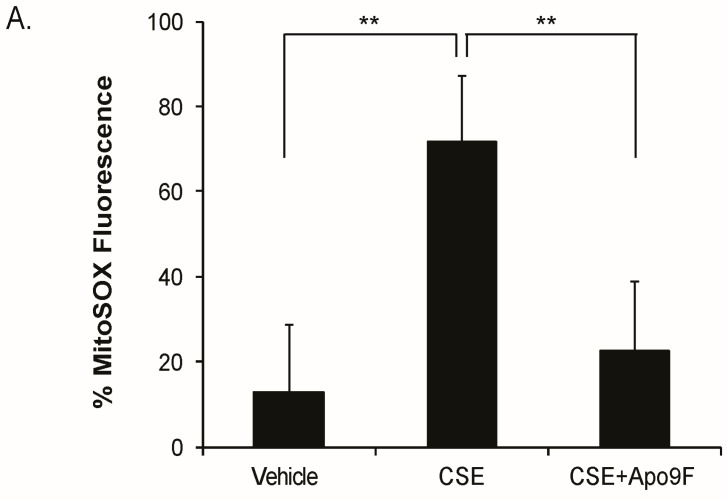

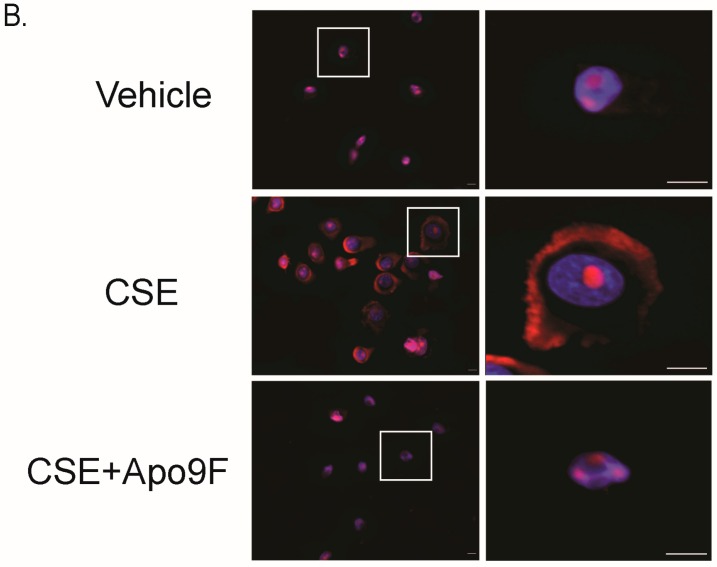
Apo9F decreases mitochondria-derived ROS in cigarette smoke-exposed HBEC2 cells. (**A**) HBEC2 cells were cultured with Apo9F (50 µM) in the presence or absence of 2% CSE for 24 h and were determined mitochondrial ROS levels. Data are expressed as mean ± SEM for three independent experiments (** *p* < 0.01); (**B**) HBEC2 cells were treated as in (**A**). Representative pictures are shown (Bars = 50 µm).

Mitochondria are the major source of ROS production in CSE-exposed lung epithelial cells [27]. To determine the effects of Apo9F on mitochondrial ROS in CSE-exposed HBEC2 cells, we cultured HBEC2 cells with Apo9F (50 μM) in the presence or absence of a lower, nonlethal dose of 2% CSE for 24 h and determined the number of cells producing mitochondrial ROS. Apo9F significantly decreased mitochondrial ROS with only 20% MitoSox-positive cells compared with 70% MitoSox-positive cells in the vehicle-treated controls (Figure 3).

### 2.4. Apo9F Attenuates Cigarette Smoke-Induced DNA Damage in HBEC2 Cells

**Figure 4:**
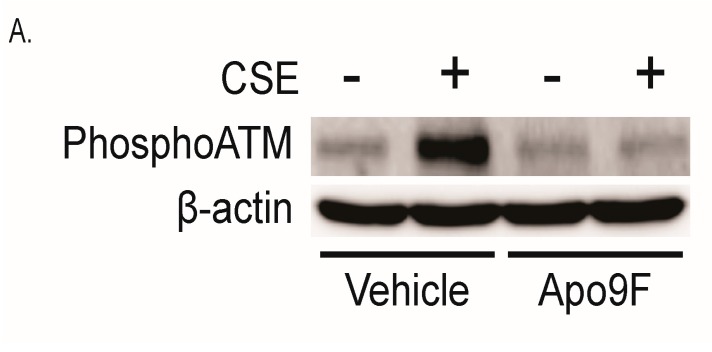

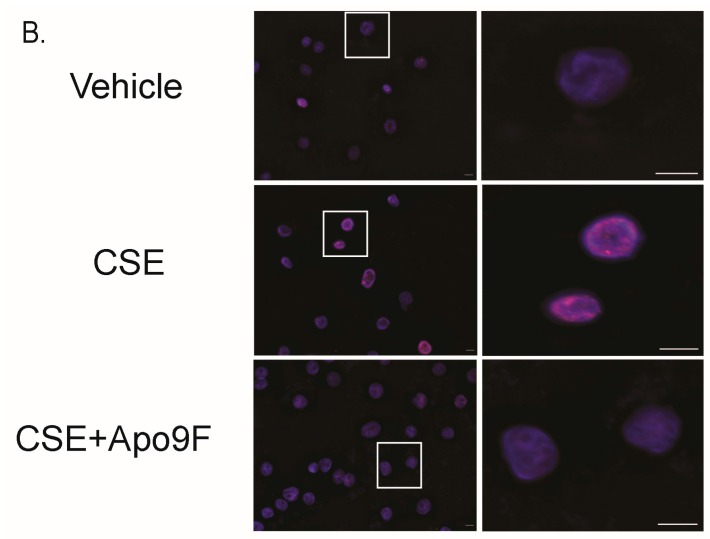
Apo9F attenuates cigarette smoke-induced DNA damage in HBEC2 cells. (**A**) HBEC2 cells were cultured with Apo9F (50 µM) in the presence or absence of 2% CSE for 24 h. Immunoblot analysis was performed for phosphorylation of ATM. Immunoblotting data are representative of three experiments; (**B**) HBEC2 cells were treated as in (**A**) and ICF (immunocytofluorescence) analysis was performed for phosphorylation of ATM. Representative pictures are shown (Bars = 50 µm).

CS exposure induces DNA damage mediated by oxidative stress [16]. Given the suppressive effects of Apo9F on mitochondrial ROS, we hypothesize that Apo9F decreases CSE-induced DNA damage. To test this, we cultured HBEC2 cells with Apo9F (50 μM) in the presence or absence of 2% CSE for 24 h. Immunoblot analysis was performed for determining the phosphorylation of ATM as a marker for DNA double strand breakage. Apo9F markedly attenuated detectable levels phospho-ATM in immunoblots from CSE-exposed HBEC2 cells (Figure 4A). To localize the DNA damage, we used immunocytofluorescence for phosphorylation of ATM and find punctate nuclear staining to be prominently induced in vehicle control/CSE-exposed cells with significant reduction of ATM phosphorylation by Apo9F pretreatment (Figure 4B).

**Figure 5:**
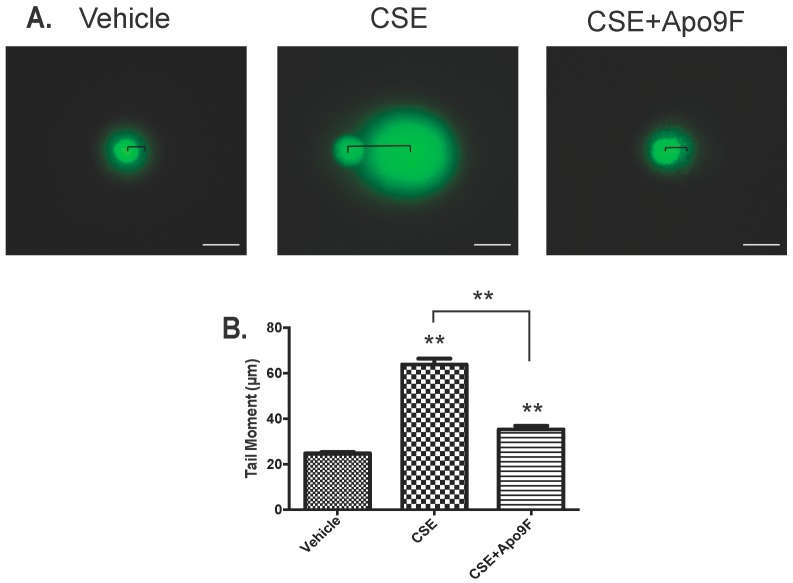
Apo9F attenuates cigarette smoke-induced DNA damage in HBEC2 cells. HBEC2 cells were cultured with Apo9F (50 µM) in the presence or absence of 5% CSE for 4 h. (**A**) Representative images from the comet assay performed to measure DNA fragmentation by loss of nuclear DNA cohesion in the “tail moment” (black bars) with fluorescent microscopic imaging; (**B**) quantitative analysis of tail moment from at least 50 individual cells from each of the three groups (** *p* < 0.01). (white scale bar, 50 µm).

We next investigated CSE-induced DNA damage and the effect of Apo9F on this phenomenon using a comet assay. In the setting of CSE exposure, HBEC2 cells display considerable DNA fragmentation and dissociation of nuclear integrity as shown by the trailing of DNA behind the main nuclear mass in an electrophoretic field (Figure 5A). The length of this “comet tail” is a robust indicator of DNA damage (Olive and Barnath Nature protocols 2006). Apo9F almost completely protects against the DNA fragmentation caused by 4 h of CSE exposure (Figure 5A,B). These data show that Apo9F attenuates DNA damage in CSE-exposed HBEC2 cells and support the finding that ATM phosphorylation after CSE exposure is a marker for DNA damage, which can be rescued by the Apo9F chemical.

## 3. Discussion

In this study, we observed that among nine marine compounds isolated from various brown algae species, Apo9F provided protection against CSE-induced apoptotic cell death in cultured HBECs. These cytoprotective effects of Apo9F were accompanied by decreases in DNA damage and mitochondrial ROS production. These results suggest that Apo9F attenuates the toxic effects of CS through mitigation of mitochondrial ROS and DNA stabilization.

Natural compounds isolated from marine products compared with land plants have been relatively understudied for potential pharmacologic utility. Marine algae represent an abundant resource for a variety of products, with over 7.5 million tons of biomass either harvested from oceans or manufactured by industries annually. About 10% of these marine algae are being utilized by some industries to generate polysaccharides, such as carrageenan, agar and alginate [28,29]. Some brown algae, such as *E. cava*, *I. foliacea*, *I. okamura*, *H. fusiformis*, and *U. peteseniana*, are edible and abundant in the oceans surrounding the Southern Korean peninsula and Japanese islands. These brown algae have long been consumed as favorable health foods among Asians and Europeans. Some compounds isolated from the brown algae have been evaluated as pharmaceuticals. For example, the phlorotannins have been reported to counteract oxidative stress and inflammation perhaps because of various molecular properties such as abundant hydroxyl bonds [30,31,32]. β-sitosterol, a marine algae-derived sterol, belongs to a class of triterpenoid lipids and has been reported to improve hypercholesterolemia in humans [33]. In addition, some algal sterols possess beneficial effects on oxidative stress [34] and inflammation [35,36], and potentially on some diseases, including cancer, diabetes [32,37], mycobacterial infections [38] and hypertension [39].

In this study, we found that the brown algae-derived compound Apo9F, which is derived from fucoxanthine through permanganate oxidation, exhibits the significant protection against CSE-induced cytotoxicity in vitro. Apo9F has been reported to possess an anti-inflammatory activity in vitro [40]. Yang et al. demonstrated that Apo9F decreases LPS (lipopolysaccharide)-induced production of nitric oxide and prostaglandin E2 with stabilization of IκB-α and thus suppression of NF-κB-mediated inducible nitric oxide synthase and cyclooxygenase-2 expression in RAW 264.7 cells [40]. Furthermore, another in vitro study revealed that Apo9F inhibits microbial DNA-induced inflammatory response by attenuating the activation of extracellular signal-regulated kinase [41].

In this study, we report a unique activity of Apo9F as an antioxidant that suppresses the accumulation of mitochondrial ROS and attenuates CSE-induced apoptosis and DNA damage in cultured human bronchial epithelial cells. These cells may model epithelia important for the development of the chronic bronchitic and asthmatic variants of COPD, which may undergo apoptosis after exposure to CS through an altered cell signaling in COPD [42,43]. While apoptosis of alveolar pneumocytes is known to be important for the development of emphysematous COPD [44,45], we speculate that CS exposure causes a similar apoptotic response in the bronchial epithelial cells [42,46].

Interestingly, previous in vitro studies supported an antioxidant activity of brown algae. Yang et al. have demonstrated that aqueous extracts from brown algae protect against H_2_O_2_-induced DNA damage and reverted the H_2_O_2_-induced cytotoxicity in H1299 cells [47].

Wen et al. also reported that the brown algae down regulates intracellular ROS, nitrogen oxide, and malonic dialdehyde (MDA) levels through upregulating the level of antioxidant enzymes such as manganese superoxide dismutase and glutathione peroxidase [48]. Apo9F may account for some amount of this antioxidant activity from brown algae.

Further studies will be required to identify more specific molecular mechanisms of protective effects of Apo9F on CSE-induced mitochondrial ROS production and evaluate the efficacy of Apo9F on CS-induced DNA damage and emphysema in vivo.

While the primary focus to prevent COPD among smokers should be smoking cessation, DNA damage in the lungs of COPD patients may persist after quitting smoking [49]. We propose that chemopreventive compounds that blunt apoptotic cell death, such as Apo9F, may prove effective for ex-smokers at risk for persistent DNA damage to halt progression of COPD lung and could also be helpful for environmental tobacco smoke-exposed nonsmokers.

## 4. Materials and Methods

### 4.1. Chemicals, Reagents, and Antibodies

Chemicals were purchased from Sigma Aldrich (St. Louis, MO, USA) and Calbiochem (La Jolla, CA, USA); Proteinase inhibitor was from Roche Life Science (Indianapolis, IN, USA). Phosphorylation of anti-ATM (serine 1981) antibody was from Cell Signaling Technology (Danvers, MA, USA) and anti-β actin was from Sigma Aldrich.

### 4.2. Natural Marine Compounds Isolated from the Brown Algae

6,6-bieckol (BK), dieckol (DK), phlorofucofuroeckol A (PFFA), phloroglucinol 6,6-bieckol (PGB), and 2,7-phyrogalyol-6,6-bieckol (2,7PGB) were isolated from *Ecklonia cava*; octaphlorethol A (OPA), diphlorethohydroxycarmalol (DPHC), saringosterol acetate (SA), and Apo9F were isolated from *Ishige foliacea*, *Ishige okamura*, *Hizikia fusiformis*, and *Undariopsis peteseniana*, respectively (Table 1). Structures of the listed compounds were determined using liquid chromatography-electrospray ionization-mass spectrometry (LC-ESI-MS, Finnigan MAT, San Jose, CA, USA), infrared spectroscopy, and nuclear magnetic resonance (NMR, JEOL JNM-LA 300, Tokyo, Japan) spectroscopy, as we previously described [20,21,22,23].

### 4.3. Cigarette Smoke Extract Preparation

Research cigarettes (3R4F) from the University of Kentucky were purchased and used to make CSE solutions. CSE solutions were prepared as we previously described [50].

### 4.4. Cell Culture and Cell Viability

Immortalized human bronchial epithelial cells (HBEC2 and BEAS-2B) were cultured and maintained as we previously described [50]. Experiments were performed in 12-well Costar tissue culture plates or 100 mm culture dishes at a starting cell density of 10 × 10^3^/cm^2^. Cells were counted with an electric particle counter (Beckman Coulter, Indianapolis, IN, USA).

Cell viability was determined by measuring the reduction of 3-(4,5-dimethylthiazol-2-yl)-2,5-diphenyl tetrazolium bromide (MTT) as we previously described (27). HBEC2 cells were cultured in 12-well plates for 24 h. Twenty-four hours later, the cells were treated with various concentrations of the 9 individual marine compounds (0, 5, 10, 25, and 50 μM; dissolved in DMSO (Sigma Aldrich) for 24 h. DMSO was used as the vehicle control at the highest volume used for the individual chemical treatment. After treatment, the cells were exposed to various concentrations of CSE for 24 h. Absorbance was measured at 540 nm. The relative cell viability of CSE-exposed cells was determined by comparing the vehicle control cells unexposed to CSE (regarded as 100% viability).

### 4.5. Flow Cytometric Analysis of Apoptotic Cells

Flow cytometry with dual staining of Annexin V and PI was performed as we previously described [26]. Briefly, following 24 h CSE exposure, cells were harvested by trypsinization. Approximately 10^5^ cells were stained in 1× binding buffer (0.01 M HEPES, pH 7.4; 0.14 M NaCl; 0.25 mM CaCl_2_) using 5 μL of Annexin V-FITC (BioLegend, San Diego, CA, USA) and 10 μL PI (BioLegend). The cells were then incubated in the dark for 15 min at room temperature and the percentage of FITC- and PI-positive cells were quantified using FACS Canto-II flow cytometer (BD Biosciences, San Jose, CA, USA) and were analyzed using FlowJo software (version 7.6.3; TreeStar, San Carlos, CA, USA).

### 4.6. Immunoblot Analysis

Immunoblot analysis was performed as we previously described [51]. Briefly, each sample was normalized for all comparisons using equivalent amounts of total protein from all adherent cells retrieved. Equivalent loading was verified by stripping the blot and reprobing with antibodies to β-actin. Results are expressed as the relative densitometry ratio (targeted protein/β-actin). The vehicle control in the absence of CSE was set to a value of 1.0.

### 4.7. Immunocytofluorescence (ICF)

Immunocytofluorescent analysis was performed as previously described [52]. Twenty-four hours after culture in the presence or absence of 50 μM Apo9F on Lab-Tek-II 8-chamber slides (Nalge Nunc International, Rochester, NY, USA), HBEC2 cells were further cultured with or without 1.5% CSE for another 24 h. Cells were fixed with 2% (*w*/*v*) paraformaldehyde in PBS for 15 min at 37 °C. The cells were then incubated with 0.2% Triton X-100 with 0.2% Saponin in a blocking solution containing 3% IgG-free BSA (bovine serum albumin), 1% Gelatin and 2% normal donkey serum for 1 h at RT (room temperature) and further incubated with the following primary antibodies at 4 °C overnight: phosphorylation-specific antibody for ATM. The immunolabeled cells were detected using F(ab)2-fragments of respective secondary antibody conjugated to DylightTM-549 (Jackson ImmunoResearch, West Grove, PA, USA) and mounted with 4′,6-diamidino-2-phenylindole (DAPI) containing Fluormount-G™ (SouthernBiotech, Birmingham, AL, USA) for nuclear staining.

### 4.8. Mitochondrial Reactive Oxygen Species

For measurement of mitochondria-derived ROS, the MitoSOX Red mitochondrial superoxide indicator (Molecular Probes, Eugene, OR, USA) was used according to the manufacturer’s instruction.

### 4.9. DNA Damage Comet Assay

The comet assay to detect DNA damage was conducted using the OxiSelect Comet assay kit (Cell Biolabs, San Diego, CA, USA) according to the manufacturer’s instructions as previously described [53,54]. In brief, four hours after 5% CSE exposure in the presence or absence of Apo9F, HBEC2 cells were harvested and suspended at a density of 750 cells/well, incubated with liquefied OxiSelect™ (Cell Biolabs) comet agarose at 1:10 ratio, transferred into the OxiSelect™ Comet slide (3 wells). The slides were immersed in pre-chilled lysis buffer for 45 min at 4 °C in the dark, transferred into pre-chilled alkaline buffer for 30 min at 4 °C, and then electrophoresed in chilled alkaline buffer at 20 V for 30 min. After washed in chilled distilled water 3 times, the cells were fixed in 70% ethanol. Once slides were dried, cells were stained with the Vista Green DNA dye. Images were captured at 40× magnification with a ZEISS Observer A1 and quantified using a software Zen 2 (ZEISS, Thornwood, NY, USA). DNA damage was quantified by measuring the tail moment. More than 50 tailed cells were analyzed per group.

### 4.10. Statistical Analysis

One-sample student unpaired *t*-test was performed for two-group comparison. For all other comparisons involving multiple treatment groups, one-way analysis of variance was used to identify treatment effects. The *p* values, based on calculated comparisons, were used to assess the individual treatment effects and were regarded as significant when the value was less than 0.05. Data were expressed as mean ± SEM.

## 5. Conclusions

In this study, our screening test using nine brown algae derived compounds revealed that apo9F exhibits robust protection against cigarette smoke-induced cytotoxicity in cultured human bronchial epithelial cells. Furthermore, apo9F prevents cigarette smoke-induced apoptosis, DNA damage, and mitochondria-derived ROS production. This highlights the potential for algal compounds as therapeutic agents in diseases associated with cell death like COPD.

## Figures and Tables

**Table 1:** Nine screened compounds isolated from the brown algae.

Species	Structure Name	Abbreviation	Molecular Weight	References for the Extraction Methods
*Ecklonia cava*	6,6-bieckol	BK	742	
	Dieckol	DK	742	
	Phlorofucofuroeckol A	PFFA	602	[20,21]
	Phloroglucinol 6,6-bieckol	PGB	972	
	2,7-phyrogalyol-6,6-biekol	2,7PGB	972	
*Ishige foliacea*	Octaphlorethol A	OPA	993	[22]
*Ishige okamura*	Diphlorethohydroxycarmalol	DPHC	512	[23]
*Undariopsis peteseniana*	Apo-9′-fucoxanthinone	Apo9F	266	[24]
*Hizikia fusiformis*	Saringosterol acetate	SA	470	[25]

## Abbreviations

The following abbreviations are used in this manuscript:

COPD	chronic obstructive pulmonary disease
CS	cigarette smoke
CSE	cigarette smoke extract
Apo9F	apo-9′-fucoxanthinone
RONS	reactive oxygen/nitrogen species
HBEC	human bronchial epithelial cells
DDR	DNA damage response
PIKKs	phosphoinositide 3-kinase related protein kinases
ATM	ataxia teleangiectasia mutated
DSB	DNA double-strand break
DK	dieckol
PFFA	phlorofucofuroeckol
PGB	phloroglucinol 6,6-bieckol
2,7PGB	2,7-phyrogalyol-6,6-bieckol
OPA	octaphlorethol
DPHC	diphlorethohydroxycarmalol
SA	saringosterol acetate
LC-ESI-MS	liquid chromatography-electrospray ionization-mass spectrometry
NMR	nuclear magnetic resonance
MTT	3-(4,5-dimethylthiazol-2-yl)-2,5-diphenyl tetrazolium bromide
DSMO	dimethyl sulfoxide
PI	propidium iodide
FITC	fluorescein isothiocyanate
FACS	fluorescence-activated cell sorting
ICF	immunocytofluorescence
DAPI	4′,6-diamidino-2-phenylindole
MDA	malonic dialdehyde
